# Association between weight-adjusted waist index and bone mineral density in adolescents

**DOI:** 10.1038/s41598-024-66565-1

**Published:** 2024-07-17

**Authors:** Guoliang Ma, Bo Xu, Dian Zhang, Liguo Zhu, Yili Zhang, Bowen Yang, Xiaokuan Qin, He Yin, Xu Wei

**Affiliations:** 1 Wangjing Hospital, China Academy of Traditional Chinese Medicine, No. 6, Zhonghuan South Road, Chaoyang District, Beijing, 100102 China; 2 Beijing Key Laboratory of Bone Setting Technology of Traditional Chinese Medicine, Beijing, 100700 China; 3 School of Integrated Chinese and Western Medicine, Nanjing University of Chinese Medicine, Nanjing, 210023 China

**Keywords:** Endocrinology, Medical research

## Abstract

The negative effects of obesity and excess body fat on bone mineral density (BMD) have been widely reported. As opposed to waist circumference (WC) or body mass index (BMI), weight-adjusted waist index (WWI) is a superior method for assessing obesity. WWI also indicates centripetal obesity independently of the weight of the individual. An investigation of WWI and adolescents’ BMD was conducted in this study. The National Health and Nutrition Examination Survey (NHANES) 2011–2018 provided the data for this cross-sectional investigation. In this study, weighted multivariate logit models were employed to assess the correlation between teenage BMD and WWI. Additionally, we conducted interaction tests and subgroup analysis. Through multivariate linear regression, we discovered that WWI was negatively linked with lumbar, trunk, and total BMD but not pelvis BMD in this study, which included 6828 subjects. We found that each unit increase in WWI resulted in a lumbar BMD decline of 0.04 g/cm^2^ (95%CI −0.04, −0.04), a trunk BMD decrease of 0.03 g/cm^2^ (95%CI −0.03, −0.02), and a total BMD decrease of 0.02 g/cm^2^ (95%CI −0.02, −0.02). In conclusion, in US teenagers, there were negative connections discovered between WWI and lumbar, trunk, and total BMD, but not pelvis BMD.

## Introduction

The medical condition known as osteoporosis, which is defined by a loss of bone mineral density, has spread throughout the world^1^, with older women and men being the main groups affected^2^. Study shows a strong relationship between bone mineral density (BMD) acquired at the end of growth and development and future osteoporosis. On the other hand, osteoporosis in adolescents is now quite well-reported^3–5^. Therefore, monitoring BMD in adolescents is important for preventing osteoporosis, identifying fracture risk, and making treatment decisions. Over the last four decades, global obesity rates have skyrocketed^6^. Previous research has established a positive association between obesity and BMD^7,8^. However, most of its parameters reflecting obesity are body mass index (BMI) and waist circumference (WC). The BMI, an indirect measure of body fat, and its use with WC has been demonstrated to have numerous limits and flaws as an indication of obesity^9^. Weight-adjusted waist index (WWI) has a stronger association with visceral obesity in comparison to BMI, therefore serving as a novel measure for evaluating obesity^10^. Numerous investigations have demonstrated a link between WWI and the likelihood of acquiring abdominal aortic calcification^11^, hepatic fibrosis^12^, and kidney stones^13^. Nevertheless, there has been no research establishing a connection between WWI and adolescent lumbar, pelvis, and trunk BMD. To ascertain whether WWI and teenage BMD are related and identify risk factors for osteoporosis and fractures in adolescence and the future, a cross-sectional analysis was conducted utilizing data obtained from the National Health and Nutrition Examination Survey (NHANES) from 2011 to 2018. We hypothesized that WWI level negatively correlated with BMD.

## Methods

### Study design and setting

**Figure 1:**
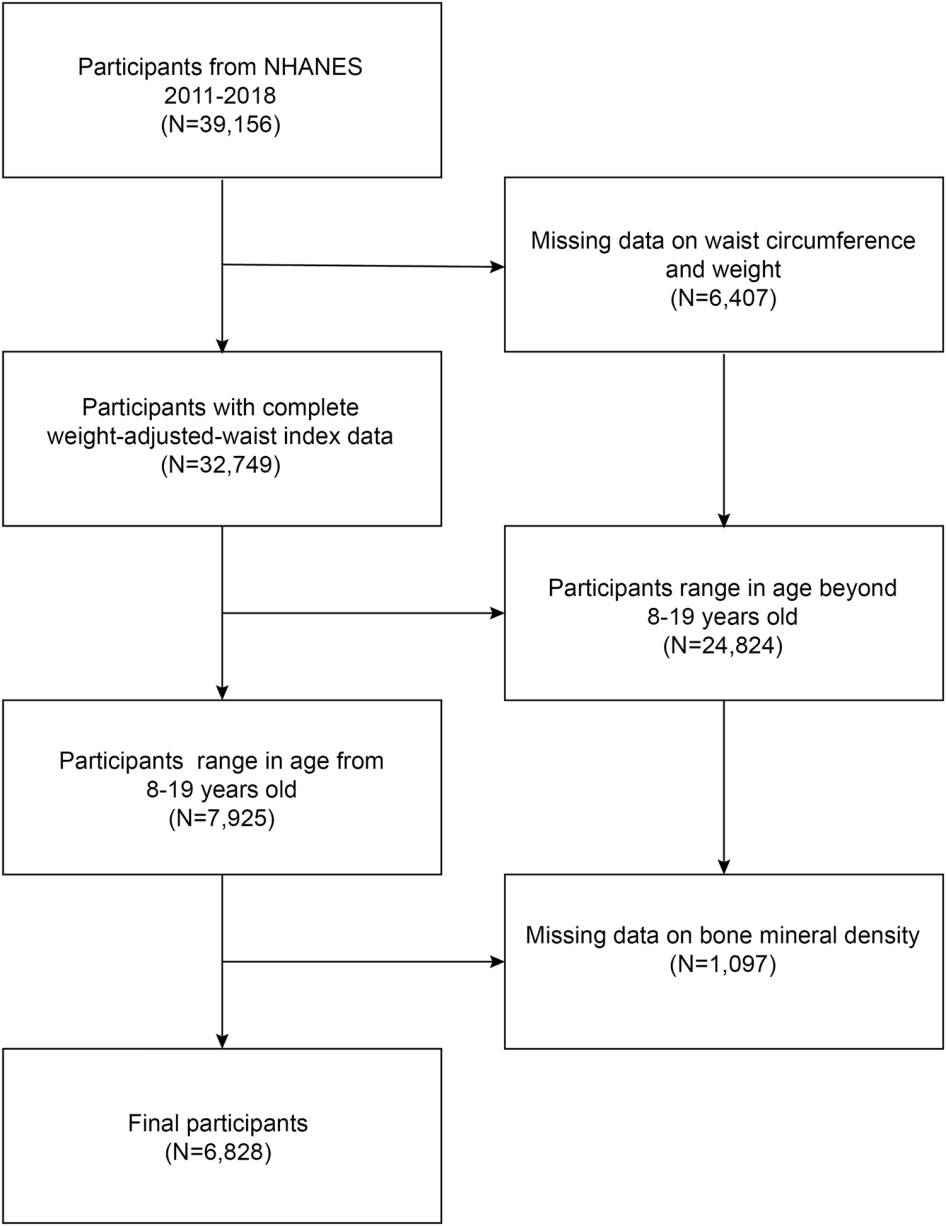
Flow chart of study population selection.

Data for this investigation were obtained from the National Health and Nutrition Examination Survey (NHANES). The National Center for Health Statistics conducts a national study called NHANES, which is based on population health and nutrition^14^. Ethics review board approval was obtained for the study from the National Center for Health Statistics Research (NCHS). The recruitment process involved obtaining the written consent of each participant^15^. Details of the Bioethics Committee number and date of consent are provided in Supplementary 1. Therefore, informed consent has been obtained from all subjects. All research was conducted in conformity with applicable guidelines/regulations. The NHANES Mobile Examination Center (MEC) conducts physical examinations on participants and collects biological samples, including blood and urine^16^. Individuals aged 12 years and above who were allocated to a morning session were instructed to abstain from eating for a duration of 9 h. The MEC phlebotomist evaluated the participant's fasting state before taking the blood sample. In the MECs and analytical laboratories, data for the laboratory component were recorded directly into a computerized database. A comprehensive directory of NHANES details is available at http://www.cdc.gov/nhanes. We gathered information for the study from four cycles involving 39,156 participants between 2011 and 2018. For 6407 participants, weight-adjusted waist circumference index data were not accessible; A total of 24,824 participants were not 8–19 years old; and for 1097 participants, BMD-related data were not available. Following the exclusion of these subjects, the final study sample comprised 6828 participants (Fig. 1). The reporting guidelines of Strengthening the Reporting of Observational Studies in Epidemiology (STROBE) were followed in this work^17^.

### Assessment of weight-adjusted-waist index

WWI was the exposure variable. The WWI is determined by dividing body weight (kg) by the square root of waist circumference (cm), which is primarily utilized to determine muscle mass and body fat mass^18^. Assessors of health certification perform weight and waist circumference measurements on participants at the mobile medical examination clinics. For weight assessment, participants are directed to remove any footwear and cumbersome clothing. To determine the waist circumference, a horizontal line is drawn from the outermost point of the right ilium outwards, and the right mid-axillary line is subsequently plotted. Following this, a measuring tape is positioned precisely at the intersection of the two aforementioned lines^19^. In subsequent analyses, participants were categorized according to their WWI quartiles (Q1: ≤ 25th percentile, Q2: > 25 to 50th percentile, Q3: > 50 to 75th percentile, Q4: > 75th percentile), and WWI was also treated as a continuous variable.

### Assessment of lumbar, trunk, pelvis, and total bone mineral density

The ending variables were lumbar, trunk, pelvis, and total BMD obtained using dual-energy X-ray absorptiometry (DXA), performed on a Hologic Discovery Model A densitometer (Hologic, Inc., Bedford, Massachusetts) using Apex version 3.2 software^20^. The assessments were conducted by a certified and trained radiographer. A comprehensive full-body scan was conducted, and the subject's BMD was computed. Additional information about the DXA examination technique can be found in the Body Composition Procedure Manual, available on the NHANES website.

### Assessment of covariates

The procedure of picking covariates is based on rational reasoning and previous scholarly research^21,22^. Covariates included age, sex, race, ratio of family income to poverty (PIR), triglycerides, low-density lipoprotein cholesterol (LDL-C), total cholesterol, glycohemoglobin, blood urea nitrogen, creatinine, serum glucose, total calcium, diabetes status, and number of days per week with at least 60 min of physical activity. The physical activities refer to those that increased participants' heart rate and made breathe hard some of the time. While other covariates were regarded as continuous variables, diabetes, number of days per week with at least 60 min of physical activity, race, and sex were evaluated as categorical variables. Race was divided into five categories: non-Hispanic White, non-Hispanic Black, Mexican American, other Hispanic, and other. Based on the data collected from the questionnaire, responses about the status of diabetes were classified as yes, no, or borderline. Days of physical activity greater than 60 min per week are categorized as 0 to 7 days.

### Statistical analysis

Weighting of all statistical analyses was performed in accordance with NHANES recommendations. In order to account for missing covariate data, multiple imputation was employed^23^. Consider the fact that the NHANES employs a multistage, intricate probability sampling design. Using chi-square and t-tests, participant demographics were evaluated in relation to WWI quartiles. An analysis of weighted multivariate logit models was conducted to examine the linear relationship between WWI and BMD; three models were constructed to test this relationship. In Model 1, no adjustments were made for covariates. In Model 2, the three covariates of race, sex, and age are accounted for. Race, sex, age, triglycerides, LDL, total cholesterol, glycosylated hemoglobin, blood urea nitrogen, creatinine, serum glucose, total calcium, diabetes, number of days per week with at least 60 min of physical activity, and the ratio of family income to poverty (PIR) were all accounted for in Model 3. Furthermore, we classified total calcium levels along a gradient consisting of low, medium, and high levels. To look into the correlation between WWI and BMD in different subgroups, we subdivided the individuals by sex, race, age, diabetes, and total calcium and carried out the subgroup analysis. A nonlinear correlation between BMD and WWI was examined using smoothed curve fitting. Utilizing threshold effect analysis, the determination of the saturation value of the link between WWI and BMD was established. Statistical analyses were carried out using R (version 3.4.3) in combination with EmpowerStats software. All analyses were subjected to a significance level of P < 0.05.

### Ethic declarations

All NHANES protocols were approved by the National Center for Health Statistics ethics review board, and each participant provided written informed permission.

## Results

### Baseline characteristics

Following the application of the inclusion and exclusion criteria, the study enrolled 6828 individuals with an average age of 13.37 ± 3.39 years. Based on the sample, 52.19 percent were male, and 47.81 percent were female. The average WWI was 10.46 (0.84) cm/√kg for all participants, while the lumbar, trunk, pelvis, and total BMD were 0.87 (0.19) g/cm^2^, 0.78 (0.15) g/cm^2^, 1.07 (0.23) g/cm^2^ and 0.95 (0.15) g/cm^2^, respectively. In comparison to those in the lowest quartile, Participants in the highest quartile of the WWI population were more likely to be youthful, female, and Mexican American, exhibiting elevated concentrations of Serum glucose, Glycohemoglobin, triglycerides, and total cholesterol in the bloodstream, while total calcium, creatinine, PIR, height, and weight were reduced. (Table 1).

**Table 1:** Basic characteristics of participants by weight-adjusted waist index quartile.

Characteristics	Weight-adjusted-waist index				*P*-Value
	**Q1** (8.04–9.87) **N = 1,707**	**Q2**(9.87–10.44) **N = 1,707**	**Q3**(10.44–11.08) **N = 1,707**	**Q4**(11.08–15.20) **N = 1,707**	
Age(years)	15.49 ± 2.34	14.05 ± 2.99	12.51 ± 3.31	11.13 ± 3.24	< 0.0001
Sex, (%)					< 0.0001
Male	72.40	41.41	44.92	49.33	
Female	27.60	58.59	55.08	50.67	
Race/ethnicity, (%)					< 0.0001
Mexican American	8.95	13.51	17.28	23.09	
Other Hispanic	6.11	7.15	8.75	9.72	
Non-Hispanic White	53.08	56.85	53.58	50.94	
Non-Hispanic Black	21.70	12.53	10.25	7.49	
Other Race	10.16	9.95	10.14	8.76	
Diabetes, (%)					0.0003
Yes	0.09	0.48	0.56	0.52	
No	99.76	99.23	98.72	98.32	
Borderline	0.16	0.29	0.73	1.17	
Days physically active at least 60 min					< 0.0001
0	7.22	5.40	5.31	5.92	
1	4.23	5.93	4.08	4.92	
2	8.14	10.24	5.96	5.81	
3	12.85	11.99	11.92	10.03	
4	13.05	10.87	7.45	5.76	
5	16.98	19.36	15.62	15.52	
6	11.91	5.79	6.72	6.47	
7	25.61	30.41	42.93	45.57	
PIR	2.56 ± 1.61	2.58 ± 1.63	2.37 ± 1.58	2.10 ± 1.46	< 0.0001
Blood urea nitrogen, (mg/dL)	11.76 ± 3.23	11.06 ± 2.82	11.01 ± 2.57	10.85 ± 1.71	< 0.0001
Total calcium, (mg/dL)	9.62 ± 0.27	9.60 ± 0.24	9.59 ± 0.21	9.58 ± 0.18	< 0.0001
Creatinine, (mg/dL)	0.78 ± 0.16	0.70 ± 0.12	0.69 ± 0.11	0.68 ± 0.09	< 0.0001
Serum glucose, (mg/dL)	87.39 ± 9.46	88.67 ± 7.76	89.53 ± 10.87	88.57 ± 6.59	< 0.0001
Glycohemoglobin, (%)	5.22 ± 0.31	5.24 ± 0.31	5.27 ± 0.29	5.30 ± 0.26	< 0.0001
Total Cholesterol, (mg/dL)	152.41 ± 21.53	156.84 ± 22.93	157.63 ± 23.37	159.60 ± 23.29	< 0.0001
Triglyceride, (mg/dL)	63.38 ± 26.69	66.92 ± 28.58	66.35 ± 25.72	67.50 ± 24.03	< 0.0001
LDL-C, (mg/dL)	83.72 ± 15.33	86.60 ± 14.19	86.39 ± 12.48	86.28 ± 10.78	< 0.0001
Lumbar BMD, (g/cm^2^)	0.99 ± 0.16	0.91 ± 0.17	0.83 ± 0.18	0.74 ± 0.16	< 0.0001
Trunk BMD, (g/cm^2^)	0.89 ± 0.13	0.80 ± 0.13	0.74 ± 0.14	0.68 ± 0.13	< 0.0001
Pelvis BMD, (g/cm^2^)	1.18 ± 0.19	1.09 ± 0.20	1.02 ± 0.24	0.95 ± 0.22	< 0.0001
Total BMD, (g/cm^2^)	1.06 ± 0.13	0.97 ± 0.13	0.91 ± 0.15	0.85 ± 0.14	< 0.0001
Weight, (kg)	61.36 ± 13.33	56.29 ± 18.15	54.66 ± 24.55	54.17 ± 26.62	< 0.0001
Height, (cm)	169.44 ± 9.19	159.39 ± 11.18	152.04 ± 14.20	145.61 ± 14.37	< 0.0001
BMI, (kg/m^2^)	21.21 ± 3.44	21.66 ± 4.82	22.55 ± 6.60	24.17 ± 7.64	< 0.0001
Waist Circumference, (cm)	73.51 ± 7.86	75.30 ± 12.10	77.59 ± 17.13	83.28 ± 20.39	< 0.0001

Mean ± SD for continuous variables: the P value was calculated by the weighted linear regression model. (%) for categorical variables: the P value was calculated by the weighted chi-square test.

*Q* quartile, *PIR* ratio of family income to poverty, *BMI* body mass index, *LDL-C* low-density, lipoprotein cholesterol.

### Association between WWI and BMD

Table 2 illustrates the link between the BMD and WWI. In addition to pelvis BMD, every model demonstrated a negative association between BMD and WWI. Once all covariates have been adjusted, a one-unit rise in WWI resulted in a decrease in lumbar, trunk, and total BMD. In particular, a reduction of 0.04 g/cm^2^ in lumbar BMD, 0.03 g/cm^2^ in trunk BMD, and 0.02 g/cm^2^ in total BMD was observed. In WWI, people from the highest and lowest quartiles were compared, and we observed 0.09 g/cm^2^, 0.06 g/cm^2^, and 0.04 g/cm^2^ reductions in lumbar, trunk, and total BMD, respectively.

**Table 2:** Association between weight-adjusted waist index and bone mineral density.

Exposure	Model 1[β (95%CI)]	Model 2[β (95%CI)]	Model 3[β (95%CI)]
Lumbar BMD (continuous)	−0.11 (−0.12, −0.11)	−0.04 (−0.05, −0.04)	−0.04 (−0.04, −0.04)
Lumbar BMD (quartile)			
Quartile 1	Reference	Reference	Reference
Quartile 2	−0.08 (−0.09, −0.07)	−0.05 (−0.05, −0.04)	−0.04 (−0.05, −0.03)
Quartile 3	−0.17 (−0.18, −0.15)	−0.06 (−0.07, −0.06)	−0.06 (−0.07, −0.05)
Quartile 4	−0.25 (−0.26, −0.24)	−0.09 (−0.10, −0.08)	−0.09 (−0.09, −0.08)
* p* for trend	< 0.0001	< 0.0001	< 0.0001
Pelvis BMD(continuous)	−0.11 (−0.11, −0.10)	−0.01 (−0.01, −0.00)	−0.00 (−0.01, 0.00)
Pelvis BMD(quartile)			
Quartile 1	Reference	Reference	Reference
Quartile 2	−0.09 (−0.10, −0.08)	−0.02 (−0.03, −0.01)	−0.01 (−0.02, −0.00)
Quartile 3	−0.16 (−0.18, −0.15)	−0.02 (−0.03, −0.01)	−0.01 (−0.02, 0.00)
Quartile 4	−0.24 (−0.25, −0.22)	−0.02 (−0.03, −0.01)	−0.01 (−0.02, 0.00)
* p* for trend	< 0.0001	0.0109	0.1584
Trunk BMD(continuous)	−0.09 (−0.10, −0.09)	−0.03 (−0.03, −0.03)	−0.03 (−0.03, −0.02)
Trunk BMD(quartile)			
Quartile 1	Reference	Reference	Reference
Quartile 2	−0.09 (−0.10, −0.08)	−0.04 (−0.05, −0.03)	−0.03 (−0.04, −0.03)
Quartile 3	−0.15 (−0.16, −0.14)	−0.05 (−0.06, −0.04)	−0.04 (−0.05, −0.04)
Quartile 4	−0.21 (−0.22, −0.20)	−0.07 (−0.07, −0.06)	−0.06 (−0.07, −0.05)
* p* for trend	< 0.0001	< 0.0001	< 0.0001
Total BMD(continuous)	−0.09 (−0.10, −0.09)	−0.02 (−0.03, −0.02)	−0.02 (−0.02, −0.02)
Total BMD(quartile)			
Quartile 1	Reference	Reference	Reference
Quartile 2	−0.09 (−0.10, −0.08)	−0.03 (−0.04, −0.03)	−0.03 (−0.03, −0.02)
Quartile 3	−0.15 (−0.16, −0.14)	−0.04 (−0.05, −0.03)	−0.03 (−0.04, −0.03)
Quartile 4	−0.21 (−0.22, −0.20)	−0.05 (−0.06, −0.04)	−0.04 (−0.05, −0.04)
* p* for trend	< 0.0001	< 0.0001	< 0.0001

Model 1: no covariates were adjusted. Model 2: age, sex, and race were adjusted. Model 3: age, sex, race, diabetes, days physically active at least 60 min, PIR, blood urea nitrogen, total calcium, creatinine, serum glucose, glycohemoglobin, total cholesterol, triglyceride, and LDL-C were adjusted.

*PIR*.ratio of family income to poverty, *LDL-C* low-density lipoprotein cholesterol.

### Subgroup analysis

In all three sites, there were inconsistent results in relation to WWI and BMD among subgroups. The association between WWI and BMD in the lumbar varied considerably between the sex-stratified subgroup analyses (interaction P < 0.0001). However, neither trunk nor total BMD showed significant differences (interaction P > 0.05). There was no statistically significant difference observed in the link between WWI and BMD across the three subgroups: diabetes, total calcium, and race. (interaction P > 0.05). Differences were found to be significant in the correlations between WWI and all BMD in age-stratified subgroup analyses. Lumbar BMD decreased by 0.05 g/cm^2^ in the 11–13 year-old age group for each unit increase in WWI, whereas it decreased by 0.02 g/cm^2^ in the 14–16 year-old age group for each unit increase in WWI. (Table 3).

**Table 3:** Subgroup analysis of the association between weight-adjusted waist index and bone mineral density.

Subgroup	Lumbar BMD [β(95%CI)]	*p* for interaction	Trunk BMD [β(95%CI)]	*p* for interaction	Total BMD [β(95%CI)]	*p* for interaction
Sex		< 0.0001		0.6644		0.5518
Male	−0.03 (−0.04, −0.03)		−0.02 (−0.03, −0.02)		−0.02 (−0.02, −0.01)	
Female	−0.05 (−0.05, −0.04)		−0.03 (−0.03, −0.02)		−0.02 (−0.02, −0.01)	
Race/ethnicity		0.6585		0.5531		0.7264
Mexican American	−0.04 (−0.05, −0.03)		−0.02 (−0.03, −0.02)		−0.02 (−0.02, −0.01)	
Other Hispanic	−0.04 (−0.05, −0.03)		−0.02 (−0.04, −0.01)		−0.02 (−0.03, −0.01)	
Non-Hispanic White	−0.04 (−0.05, −0.03)		−0.03 (−0.03, −0.02)		−0.02 (−0.02, −0.02)	
Non-Hispanic Black	−0.03 (−0.04, −0.02)		−0.02 (−0.03, −0.01)		−0.02 (−0.02, −0.01)	
Other Race	−0.05 (−0.06, −0.03)		−0.03 (−0.04, −0.02)		−0.02 (−0.03, −0.01)	
Diabetes		0.6978		0.6609		0.4847
Yes	−0.05 (−0.25, 0.14)		−0.04 (−0.21, 0.12)		−0.02 (−0.18, 0.13)	
No	−0.04 (−0.04, −0.03)		−0.03 (−0.03, −0.02)		−0.02 (−0.02, −0.02)	
Borderline	-0.00 (-0.09, 0.08)		0.00 (-0.06, 0.07)		0.02 (-0.04, 0.08)	
Age		< 0.0001		< 0.0001		0.0016
8–10 years old	−0.03 (−0.04, −0.02)		−0.02 (−0.02, −0.01)		−0.01 (−0.02, −0.01)	
11–13 years old	−0.05 (−0.05, −0.04)		−0.03 (−0.03, −0.02)		−0.02 (−0.03, −0.02)	
14–16 years old	−0.02 (−0.03, −0.01)		−0.01 (−0.01, 0.00)		−0.01 (−0.01, 0.00)	
17–19 years old	−0.04 (−0.05, −0.03)		−0.02 (−0.03, −0.01)		−0.01 (−0.02, −0.01)	
Total calcium		0.3036		0.7262		0.4188
7.30–9.40	−0.03 (−0.04, −0.02)		−0.01 (−0.02, −0.01)		−0.01 (−0.02, −0.00)	
9.40–9.50	−0.03 (−0.04, −0.02)		−0.02 (−0.02, −0.01)		−0.01 (−0.02, −0.01)	
9.50–11.3	−0.04 (−0.04, −0.03)		−0.02 (−0.02, −0.01)		−0.01 (−0.02, −0.01)	

Age, sex, race, diabetes, days physically active at least 60 min, PIR, blood urea nitrogen, total calcium, creatinine, serum glucose, glycohemoglobin, total cholesterol, triglyceride, and LDL-C were adjusted.

*PIR* ratio of family income to poverty, *LDL-C* low-density lipoprotein cholesterol.

### Analysis of nonlinear and saturation effects of weight-adjusted waist index and bone mineral density.

**Figure 2:**
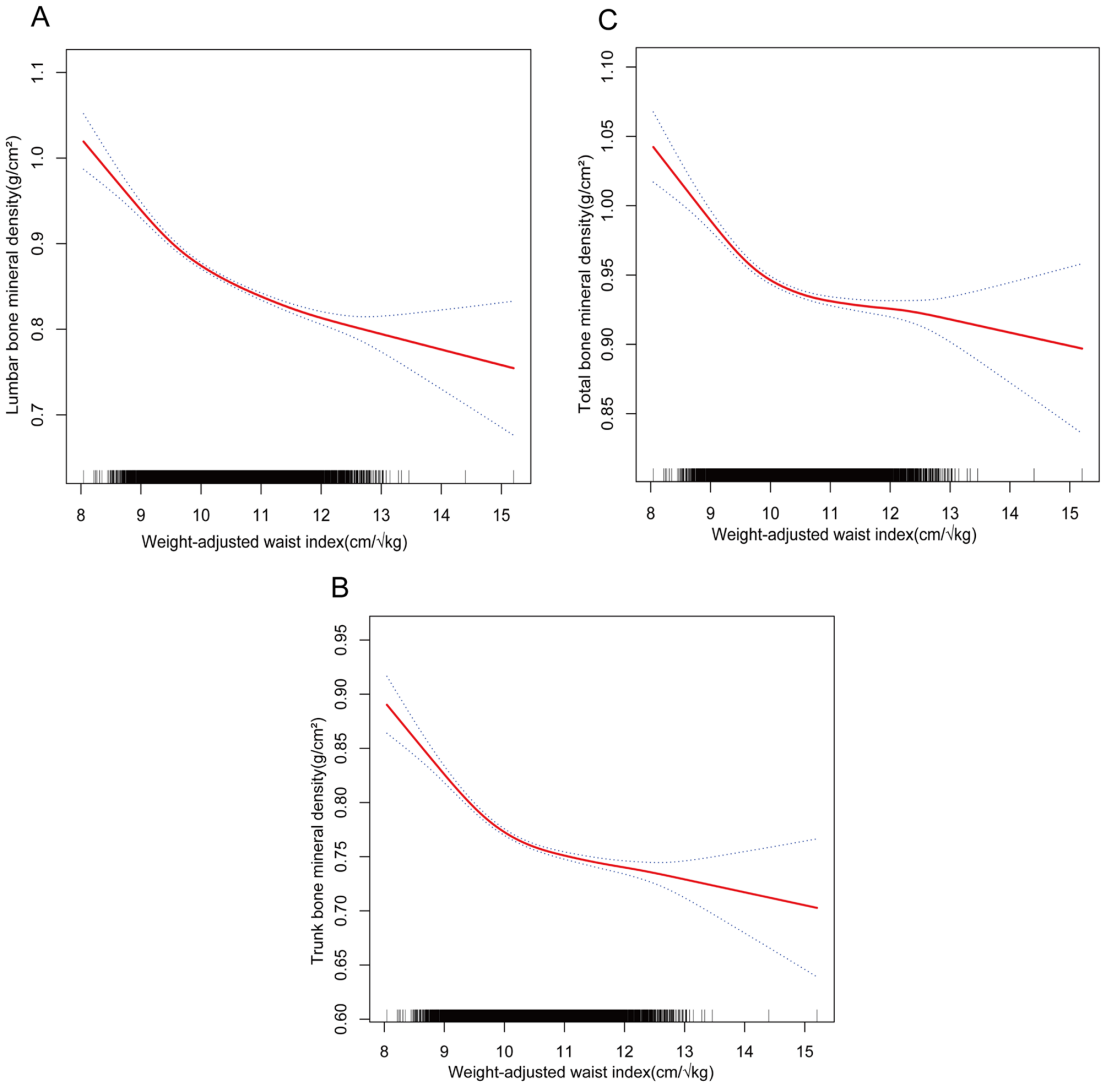
Nonlinear relationship between weight-adjusted waist index and bone mineral density. The solid red line represents a smooth curve fit between the variables. Dashed lines representing 95% confidence intervals were taken. (**A**) WWI versus lumbar BMD; (**B**) WWI versus trunk BMD; (**C**) WWI versus total BMD.

**Figure 3:**
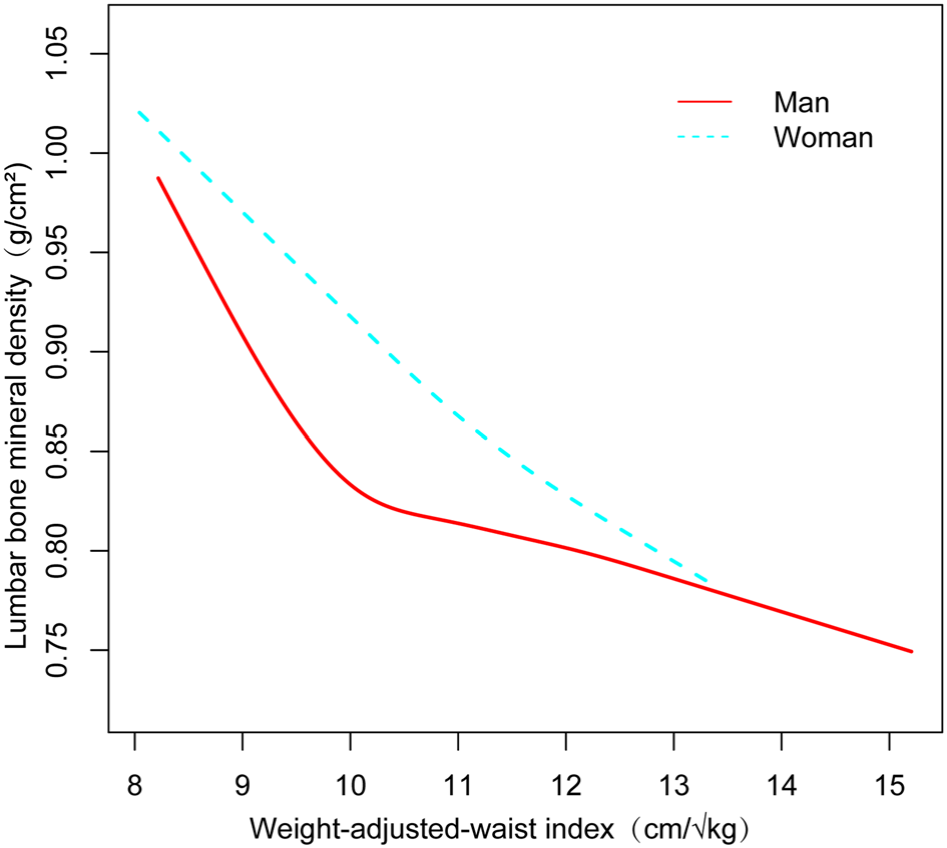
The nonlinear association between weight-adjusted waist index and lumbar bone mineral density stratified by sex.

The nonlinear relationship and saturation phenomenon between WWI and lumbar, trunk, and total BMD were shown using smoothed curve fitting (Fig. 2). According to WWI-lumbar BMD saturation effect values for all participants, the average value was 9.87 cm/√kg. In case of WWI under 9.87 cm/√kg, the effect value was recorded as − 0.08; Alternatively, when WWI surpassed 9.87 cm/√kg, the effect value changed to -0.03. WWI-trunk BMD saturation effect value for all participants was 9.95 cm/√kg. At WWI values below 9.95 cm/√kg, the effect value was recorded as -0.06. Meanwhile, when the value of WWI surpassed 9.95 cm/√kg, the corresponding effect value underwent a shift to −0.02 (Table 4). According to WWI-total BMD obtained from all participants, the saturation effect value was 9.88 cm/√kg. At WWI values beneath 9.88 cm/√kg, the effect value was−0.06; the effect value changed to −0.01 when WWI exceeded 9.88 cm/√kg. Grouping all participants by age, the WWI effect values for each group of BMDs were determined by smoothing curves and saturation effect assessments (Table 4). Participants were equally divided by age into four groups (8–10, 11–13,14–16,17–19) and the most significant saturation effects between WWI and lumbar, trunk, and total BMD were found for the 11–13 year old group. In terms of the saturation effect of WWI with lumbar BMD, for WWI < 9.97 cm/√kg, each unit increase in WWI resulted in a lumbar BMD decline of 0.12 g/cm^2^ (95%CI −0.15, −0.09); in contrast, for individuals with WWI > 9.97 cm/√kg, each unit increase in WWI resulted in a lumbar BMD decline of 0.03 g/cm^2^ (95%CI −0.04, −0.03). Regarding the connection between WWI and trunk BMD, for WWI < 9.96 cm/√kg, each unit increase in WWI resulted in a trunk BMD decline of 0.11 g/cm^2^ (95%CI −0.13, −0.08); by comparison, for individuals with WWI > 9.96 cm/√kg, each unit increase in WWI resulted in a trunk BMD decline of 0.02 g/cm^2^ (95%CI −0.02, −0.01). About the relationship between WWI and total BMD, for WWI < 9.95 cm/√kg, each unit increase in WWI resulted in a total BMD decline of 0.09 g/cm^2^ (95%CI −0.11, −0.07); by contrast, for individuals with WWI > 9.95 cm/√kg, each unit increase in WWI resulted in a total BMD decline of 0.01 g/cm^2^ (95%CI −0.02, −0.00). All participants were grouped by sex and smoothed curves and saturation effects assessment were taken to illustrate effect values for lumbar BMD with WWI (Table 5, Fig. 3). Among male participants, for WWI < 9.95 cm/√kg, each unit increase in WWI resulted in a lumbar BMD decline of 0.09 g/cm^2^ (95%CI −0.11, −0.08); in contrast, for individuals with WWI > 9.95 cm/√kg, each unit increase in WWI resulted in a lumbar BMD decline of 0.02 g/cm^2^ (95%CI −0.02, −0.01). Within the group of female participants, for WWI < 11.79 cm/√kg, each unit increase in WWI resulted in a lumbar BMD decline of 0.05 g/cm^2^ (95%CI −0.06, −0.05). However, When WWI was more than 10.29 cm/√kg, there was no statistically significant correlation between WWI and lumbar BMD (β = 0.00, 95% CI −0.03, 0.03).

**Table 4:** Saturation effects between weight-adjusted waist index and bone mineral density stratified and results stratified by age.

Lumbar BMD	Adjusted β (95%CI)	Trunk BMD	Adjusted β (95%CI)	Total BMD	Adjusted β (95%CI)
WWI turning point(K)	9.87		9.95		9.88
< K, effect1	−0.08 (−0.09, −0.07) < 0.0001		−0.06 (−0.07, −0.05) < 0.0001		−0.06 (−0.07, −0.05) < 0.0001
> K, effect2	−0.03 (−0.04, −0.03) < 0.0001		−0.02 (−0.02, −0.01) < 0.0001		−0.01 (−0.01, −0.01) < 0.0001
Log-likelihood ratio	< 0.001		< 0.001		< 0.001
Stratified by age					
WWI turning point(K) for 8-10 years old	11.34		11.34		10.73
< K, effect1	−0.04 (−0.05, −0.04) < 0.0001		−0.03 (−0.04, −0.02) < 0.0001		−0.04 (−0.06, −0.03) < 0.0001
> K, effect2	−0.01 (−0.02, 0.00) 0.0824		−0.00 (−0.01, 0.01) 0.7048		−0.00 (−0.01, 0.00) 0.1926
Log-likelihood ratio	< 0.001		< 0.001		< 0.001
WWI turning point(K) for 11-13 years old	9.97		9.96		9.95
< K, effect1	−0.12 (−0.15, −0.09) < 0.0001		−0.11 (−0.13, −0.08) < 0.0001		−0.09 (−0.11, −0.07) < 0.0001
> K, effect2	−0.03 (−0.04, −0.03) < 0.0001		−0.02 (−0.02, −0.01) < 0.0001		−0.01 (−0.02, −0.00) 0.0010
Log-likelihood ratio	< 0.001		< 0.001		< 0.001
WWI turning point(K) for 14-16 years old	9.37		9.43		9.84
< K, effect1	−0.14 (−0.20, −0.09) < 0.0001		−0.11 (−0.15, −0.07) < 0.0001		−0.05 (−0.07, −0.03) < 0.0001
> K, effect2	−0.01 (−0.02, −0.00) 0.0470		0.01 (−0.00, 0.01) 0.1846		0.01 (0.00, 0.02) 0.0226
Log-likelihood ratio	< 0.001		< 0.001		< 0.001
WWI turning point(K) for 17-19 years old	9.91		9.95		9.89
< K, effect1	−0.07 (−0.09, -0.04) < 0.0001		−0.04 (−0.06, −0.02) < 0.0001		−0.04 (−0.06, −0.03) < 0.0001
> K, effect2	−0.03 (−0.04, −0.01) 0.0002		−0.01 (−0.02, 0.00) 0.1246		0.00 (−0.01, 0.01) 0.9989
Log-likelihood ratio	0.011		0.011		< 0.001

Age, sex, race, diabetes, days physically active at least 60 min, PIR, blood urea nitrogen, total calcium, creatinine, serum glucose, glycohemoglobin, total cholesterol, triglyceride, and LDL-C were adjusted.*PIR* Ratio of family income to poverty, *LDL-C* Low-density lipoprotein cholesterol.

**Table 5:** Saturation effects between weight-adjusted waist index and lumbar bone mineral density stratified by sex.

Lumbar bone mineral density	Adjusted β (95%CI)
Stratified by sex	
Man	
WWI turning point(K)	9.95
< K, effect1	−0.09 (−0.11, −0.08) < 0.0001
> K, effect2	−0.02 (−0.02, −0.01) < 0.0001
Log-likelihood ratio	< 0.001
Woman	
WWI turning point(K)	11.79
< K, effect1	−0.05 (−0.06, −0.05) < 0.0001
> K, effect2	0.00 (−0.03, 0.03) 0.9202
Log-likelihood ratio	0.002

Age, sex, race, diabetes, days physically active at least 60 min, PIR, blood urea nitrogen, total calcium, creatinine, serum glucose, glycohemoglobin, total cholesterol, triglyceride, and LDL-C were adjusted.*PIR* Ratio of family income to poverty, *LDL-C* Low-density lipoprotein cholesterol.

## Discussion

With 6828 participants, a complex correlation between WWI and BMD in teenagers was identified through an examination of cross-sectional data. Our research showed that WWI was negatively correlated with lumbar, trunk, and total BMD, independent of pelvic BMD, suggesting that an elevated manifestation of WWI could potentially lead to a reduction in BMD. This is, as far as we are aware, the first investigation into the correlation between WWI and the BMD of the lumbar, trunk, and pelvis in adolescents. Although BMI is the most prevalent metric for assessing obesity, Its ability to distinguish between visceral or abdominal fat and muscle mass or fat is limited^24^. The WC, which is closely associated with visceral fat and serves as an indicator of abdominal obesity, has been suggested as a more precise body fat index than BMI in forecasting obesity-related ailments. Furthermore, it has been demonstrated to be practical in predicting the risk of cardiovascular disease and mortality^25,26^. Nevertheless, the measurement of waist circumference as a BMI-independent index is constrained by its high correlation with BMI and its tendency to reflect low muscle mass^10^. As an anthropometric indicator, WWI, on the other hand, there is a positive correlation between it and adipose mass, while a negative correlation exists with muscle mass^27^, which is more accurate in reflecting visceral fat and poor metabolic characterization of the body^28^. According to epidemiologic studies, predicating estimations of mortality in all causes, obesity, and cardiovascular disease on WWI data provide greater accuracy than WC and BMI^27,29–31^. As previous research mentioned, obese individuals have a considerably greater BMD and a reduced risk of fracture compared to their healthy-weight counterparts^32,33^. The phenomenon known as the “obesity paradox” has been applied to this protective effect of obesity^34^. On the contrary, research has demonstrated that body fat, particularly abdominal fat, negatively affects BMD and fracture risk^35^. In obese individuals, fractures are more likely to occur, particularly those who have a lower muscle mass and more visceral adipose tissue^36^. Certain scholars have posited that the limitation of the BMI to differentiate between muscle mass and adipose mass could account for a portion of the obesity paradox^37^.

Our study suggests that participants in the highest quartile of WWI are more likely to be youthful, females. Obesity prevalence in males showed an inverse association with moderate to vigorous physical activity (MVPA) and total activities, however, no such association was observed in youthful females. And youthful females may engage in physical activity for shorter periods of time or with less intensity than youthful males^38^. This could partially explain why the group in the highest quartile of WWI is young women and elucidate the disparities in obesity rates between male and female teenagers. The results of the multiple regression analysis showed that each model showed that WWI was negatively correlated with lumbar, trunk, and total BMD, but not with pelvic BMD. The unstable association between WWI and pelvic BMD may be attributed to the unreliability of pelvic BMD as a measurement site in comparison to spinal BMD in children and adolescents. Consequently, the accuracy and reproducibility of pelvic BMD measurements are inadequate, resulting in uncertainty regarding the association between WWI and pelvic BMD^39,40^. The results of the subgroup and saturation effects analyses indicated that the negative correlation effect between WWI and BMD, particularly lumbar BMD, was most consistently and strongly associated with the cohort of 11–13 year olds. While the precise causes remain unknown, several hypotheses can be provided. An analysis of cohort research involving 198 adolescents aged 9–19 years revealed that female participants' BMD increased particularly significantly between the ages of 11–14 years. Meanwhile, the rate of increase in lumbar BMD was much higher compared to BMD at other sites^41,42^. Consequently, the detrimental impact of WWI on BMD, particularly lumbar BMD, in this particular cohort is significantly more pronounced. Based on the subgroup analysis, there was a statistically significant difference between sex regarding the association between lumbar BMD and WWI (interaction P < 0.0001). By incorporating the data of 40,568 adults in the NHANES from 1999 to 2018 and employing a smoothed curve fitting with subgroup analysis, Zhang et al. argued that the relationship between WWI and total BMD exhibited variations based on the sex-stratified subgroup (interaction P = 0.016), but not at lumbar, pelvic, and femoral neck BMD sites^18^. Inconsistencies in results could potentially be attributed to variations in covariates and the age of the groups included.

WWI and BMD have been shown to have negative correlations, which may be attributed to the following mechanisms. In conjunction with our research, participants in the highest WWI quartile had higher blood levels of total cholesterol and LDL-C compared to those in the lowest WWI quartile. It has been demonstrated that both in vitro and in vivo settings, elevated cholesterol and its metabolites impair osteoblast functional activity^43,44^. Simultaneously, the products of LDL oxidation were capable of both inhibiting osteoblast differentiation and promoting adipocyte differentiation^45^, which ultimately results in a decrease in BMD. On the other hand, BMD may have been reduced because obesity decreases lipocalin concentrations, causing osteoclastogenesis to be stimulated and osteoblastogenesis to be inhibited^46^. Evidence suggests that chronic inflammation and enhanced pro-inflammatory cytokine levels in the bloodstream and tissues, like TNFα and IL6, are linked to obesity. Through NFκB-mediated production of the inflammatory mediator TNFα, which upregulates the expression of c-fms, RANK, and RANKL, obesity enhances osteoclastogenesis^47^. Individuals who have systemic obesity and exhibit elevated levels of peroxisome proliferator-activated receptor-γ (PPARγ) metabolites exhibit adipose infiltration into the bone marrow and diminished differentiation of the common progenitor to osteoblasts.^48^.In addition, obesity can contribute to the reduction of bone and osteoblast by inducing the differentiation of adipocytes from bone marrow mesenchymal stem cells^49^.

There are certain restrictions on our research: first, as this was a cross-sectional study, we had difficulty in determining the causal link between WWI and BMD in adolescents. Meanwhile, data limitations made it difficult for us to include all covariates that might influence bone metabolism, and we were unable to totally rule out the possibility that any confounders could have affected the outcomes. The NHANES questionnaire did not identify the type of physical activity in the covariate about the number of days per week with at least 60 min of physical activity, so there may be some limitations in clinical generalization. However, it is undeniable that our results are representative due to the large sample size and the fact that the study data were from a national sample.

## Conclusion

According to our research, there may be a negative correlation between teenage WWI and the lumbar, trunk, and total BMD, but not pelvis BMD. More precisely, there was a reduction of 0.04 g/cm^2^ in lumbar BMD, 0.03 g/cm^2^ in trunk BMD, and 0.02 g/cm^2^ in total BMD for each unit increase in WWI. Subgroup analysis results showed statistically significant sex differences between lumbar BMD and WWI. However, further extensive prospective investigations are still required to validate our findings.

### Supplementary Information

Supplementary Information.

## Data Availability

All research data for this paper can be found in the NHANES (http://www.cdc.gov/nchs/nhanes.htm).

## Abbreviations

WWI: Weight-adjusted waist index
BMD: Bone mineral density
BMI: Body mass index
WC: Waist circumference
NHANES: National health and nutrition examination survey
NCHS: National center for health statistics
LDL-C: Low-density lipoprotein cholesterol
PIR: Ratio of family income to poverty
RANK: Receptor activator of nuclear factor-κ B
RANKL: Receptor activator of nuclear factor-κ B ligand
PPARγ: Peroxisome proliferator-activated receptor-γ
BMSC: Bone marrow mesenchymal stem cell
MVPA: Moderate to vigorous physical activity
