# Supplementary figures and images for: Association between weight-adjusted waist index and bone mineral density in adolescents
Source: Sci Rep. 2024 Jul 17;14:16509. doi: 10.1038/s41598-024-66565-1 (PMC11255232; doi:10.1038/s41598-024-66565-1)

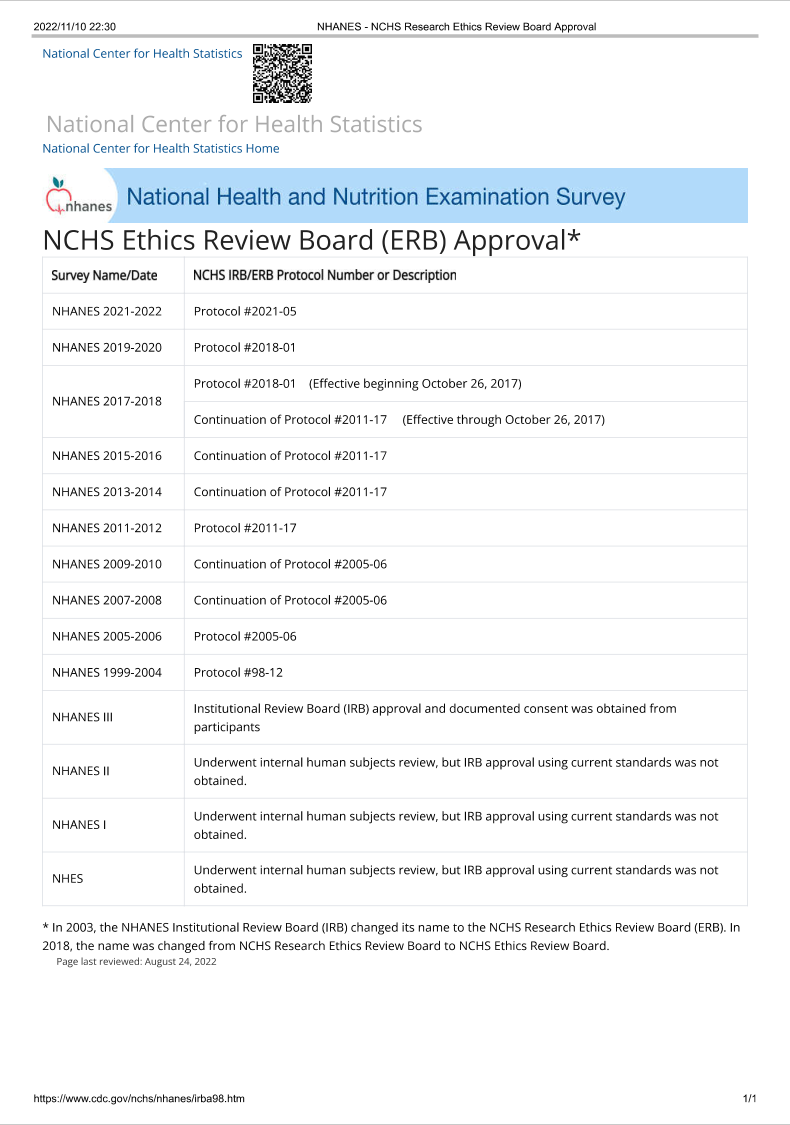

Supplement: Supplementary file 1 — Supplementary Information. [file 41598_2024_66565_MOESM1_ESM.docx]
